# Evaluation of Integrity of Allogeneic Bone Processed with High Hydrostatic Pressure: A Pilot Animal Study

**DOI:** 10.34133/bmr.0067

**Published:** 2024-08-15

**Authors:** Janine Waletzko-Hellwig, Jan-Oliver Sass, Rainer Bader, Bernhard Frerich, Michael Dau

**Affiliations:** ^1^ Department of Oral, Maxillofacial and Plastic Surgery, Rostock University Medical Center, 18057 Rostock, Germany.; ^2^ Research Laboratory for Biomechanics and Implant Technology, Department of Orthopaedics, Rostock University Medical Center, 18057 Rostock, Germany.

## Abstract

Processing of bone allografts with strong acids and γ-sterilization results in decreased biomechanical properties and reduction in osteogenecity and osteoconductivity. High hydrostatic pressure (HHP) treatment could be a gentle alternative to processing techniques usually applied. HHP is known to induce devitalization of cancellous bone while preserving biomechanical stability and molecules that induce cell differentiation. Here, a specific HHP protocol for devitalization of cancellous bone was applied to rabbit femoral bone. Allogeneic bone cylinders were subsequently implanted into a defect in the lateral condyles of rabbit femora and were compared to autologous bone grafts. Analysis of bone integration 4 and 12 weeks postoperatively revealed no differences between autografts and HHP-treated allografts regarding the expression of genes characteristic for bone remodeling, showing expression niveous comparable to original bone cylinder. Furthermore, biomechanical properties were evaluated 12 weeks postoperatively. Autografts and HHP-treated allografts both showed a yield strength ranging between 2 and 2.5 MPa and an average bone mass density of 250 mg/cm^2^. Furthermore, histological analysis of the region of interest revealed a rate of 5 to 10% BPM-2 and approximately 40% osteocalcin-positive staining, with no marked differences between allografts and autografts demonstrating comparable matrix deposition in the graft region. A suitable graft integrity was pointed out by μCT imaging in both groups, supporting the biomechanical data. In summary, the integrity of HHP-treated cancellous bone allografts showed similar results to untreated autografts. Hence, HHP treatment may represent a gentle and effective alternative to existing processing techniques for bone allografts.

## Introduction

The treatment of bone defects remains clinically challenging in various disciplines including maxillofacial surgery, orthopedics, and trauma surgery [1]. In addition to trauma or tumors, bone damages can also be caused by osteoporotic fractures [2]. In principle, bone can regenerate itself due to its improved vascularization, but if the defect exceeds a critical size, self-regenerating properties are diminished [3]. Besides this, various local and systemic factors such as blood supply, infections, smoking, obesity, or advanced age can contribute negatively to bone healing processes. In this case, surgeons have to use bone replacement materials in order to reconstruct these defects [4]. Autologous bone is still known as the clinical gold standard with the huge advantage of a low probability of rejections as donor and recipient are the same individual [5]. Furthermore, it is characterized by osteoinductive and osteoconductive properties as well as a certain osteogenicity due to the presence of proteins that promote bone remodeling processes. Nevertheless, its availability is limited for anatomical reasons and comorbidities cannot be ruled out [6]. Alternatives to autologous bone such as allogeneic materials stand out with better availability. However, these grafts have to be exposed to processing methods before implantation in order to avoid immunological rejections due to residual cellular components of the donor. The typically used cleaning processes often comprise decellularization and decontamination and include peracetic acid–ethanol treatments and gamma sterilization [7]. The main disadvantage of these treatments is the resulting reduced biomechanical properties of the graft, which make them inappropriate for load-bearing applications [8]. Furthermore, chemical processes denature proteins, which leads to a loss of osteogenic and osteoinductive properties [9]. These are essential to induce bone remodeling and the optimal integration of the graft. Therefore, a processing method should be preferred, which preserves biomechanical as well as osteoinductive and osteoconductive properties while providing an immunological harmless graft [4]. An alternative to known bone graft processing methods could be represented by high hydrostatic pressure (HHP). HHP is primarily known from the food industry, where it is used to preserve dairy products but does not influence the flavor of the products. In recent years, HHP has also gained more attention in medical- and pharmaceutical-related fields [10]. It was shown that HHP successfully devitalizes mammalian cells and, depending on the applied pressure height, can induce apoptosis or necrosis [11]. Also, human cancellous bone does react apoptotic or necrotic to HHP [12]. It was shown that the type of cell death had an influence on the differentiation of osteoclast progenitor cells and thus on bone resorption in vitro [13]*.* This can be primarily attributed to the release of immune system-activating molecules such as damage-associated molecular patterns (DAMPs), which promote resorption of the bone [14]. Since excessive resorption of bone should be avoided in order to reduce the potential of graft failure, for this in vivo study a pressure protocol using HHP below 300 MPa was chosen for which moderate resorption was observed in vitro [13]. Furthermore, applying this HHP on cancellous bone and incubating this with mesenchymal stem cells led to the promotion of osteogenic cell differentiation in vitro [15]. In addition to the specific induction of cell death, HHP processing has the big advantage where the mechanical properties of tissues are not negatively affected. This can be explained by the fact that both proteins and structure are not damaged during HHP processing as studies on bones and tendons have shown [12]. On the basis of our previous works, the presented study aims to determine whether HHP-treated bone allografts are also suitable for in vivo application using a rabbit model. Although HHP is only a devitalizing but not decellularizing process, residual cellular components are still present in the bone. Despite the fact that the DNA content cannot be reduced using HHP, cells should appear apoptotic so the immune system can react in a reduced immunological response as in vitro studies showed [16]. Considering this, an application of less than 300 MPa for processing of bone allografts was used, which led to apoptosis [17]. Furthermore, autologous bone was chosen as a benchmark to assess bone integration as this is still considered as the clinical gold standard for reconstruction of bone defects. The purpose of this study was to evaluate the degree to which HHP-processed allografts are functionally subject to autograft transplantation. Therefore, a bone defect at the lateral condyles of both femora was created, analogous to previous studies [18]. On the one side, the defect was reconstructed using the HHP-treated allograft, whereas the autograft was inserted on the opposite side as reference. After 4 and 12 weeks, the mechanical properties were examined by compression testing and the graft integration was evaluated by analysis of micro-computed tomography (μCT) images and histology. In addition, gene expression analyses of the bone grafts were conducted regarding genes involved in bone remodeling processes.

## Materials and Methods

### Sample extraction and HHP treatment

In preparation of the surgical procedure, femora were collected from female New Zealand white rabbits, which have been sacrificed in previous animal experiments at the Rostock University Medical Center (LALLF M-V/TSD/7221.3-1-076/20). Femora were cleared of surrounding soft tissue and stored at −20 °C until use. Prior to surgery, bones were slowly thawed at 4 °C and bone cylinders were harvested laterally of the epicondyles using a trepan drill (∅ 6 mm, Ustomed Instrumente, Tuttlingen, Germany). In order to prevent necrosis that could result from heat during drilling, bone was continuously rinsed with 0.9% saline solution during the removal process. Afterward, specimens were transferred into CryoTubes (Thermo Fisher Scientific, Waltham, MA, USA) filled with sterile phosphate-buffered saline (PBS; Sigma-Aldrich, Munich, Germany) and stored at 4 °C until HHP treatment.

HHP processing was performed in a customized HHP device (Dustec GmbH, Wismar, Germany) before surgery. For this purpose, the samples were transferred into fresh PBS under sterile conditions and cryotubes were sealed free of air bubbles. A pressure of up to 200 MPa was applied for 20 min at 30 °C. Bone cylinders were then removed from the medium and rinsed with 20 ml of PBS supplemented with 1% penicillin/streptomycin (Sigma-Aldrich, Munich, Germany). The allogeneic bone cylinders were stored on ice in the previously mentioned medium until implantation.

### Surgical procedure and sample collection

The animal study was approved by the local Animal Research Committee [Landesamt für Landwirtschaft, Lebensmittelsicherheit und Fischerei (LALLF)] of the state Mecklenburg-Western Pomerania (LALLF M-V/TSD/7221.3-1-076/20). All animal experiments were conducted at the Core Facility of the Central Animal Husbandry, Rostock University Medical Center, Germany. A 14-day adaptation period was allocated for the animals to acclimate to the new husbandry conditions, staff handling, and the dietary regimen before the commencement of the experiments. The quantity of feed provided was adjusted to meet the animals’ maintenance requirements and was based on their respective body weight. Water was available to the animals ad libitum. The animals were kept under a defined day/night rhythm.

In summary, 14 adult female New Zealand white rabbits (Charles River, Sulzlingen, Germany) with weight between 3.6 and 4.3 kg and an age between 18 and 26 months were used. Animals were divided into 2 groups of 6 animals sacrificed 4 weeks postoperatively and 8 animals sacrificed 12 weeks postoperatively. An overview of the used animals and the subsequent analysis of samples can be found in Table 1.

Animals were anesthetized with 50 mg/kg body weight ketamine subcutaneously (10%, bela-pharm GmbH & Co. KG, Vechta, Germany) and 5 mg/kg body weight xylazine subcutaneously (2%, Bayer AG, Leverkusen, Germany). Since both hind legs underwent a surgical procedure, 2 ml of local anesthesia lidocaine hydrochloride was administered on one side first and later on the other one (xylocitin 2%, MIBE GmbH, Brehna, Germany). Furthermore, 150,000 international units of penicillin G was administered intramuscularly to prevent microbial infections (InfectoPharm, Heppenheim, Germany). Carprofen (4 mg/kg body weight) (Pfizer Deutschland GmbH, Berlin, Germany) was also applied intramuscularly for the reduction of pain. Afterward, the hind legs were shaved lateral to the epicondyles and disinfected (Betaisodona, Mundipharma GmbH, Frankfurt, Germany). The lateral condyles were then exposed without ligamentous injury, and the periosteum was removed. Subsequently, a defect with a diameter of 6 mm and a depth of 7 to 10 mm was created using a trephine drill under continuous flushing with 0.9% saline solution. On the contralateral side, the removed bone cylinder was reintegrated randomly rotated into the defect immediately (autograft). On the other side, the trephined cylinder was replaced by an HHP-treated bone cylinder, which was obtained from sacrificed animals as previously described (allograft). Prior to implantation, the bone cylinders were equipped with a tantalum pin (∅ 0.5 mm, 4 mm in length; X-Medics Scandinavia, Frederiksberg, Denmark) to identify the defect area by μCT after sacrifice of the animals. Afterward, wounds were closed using both absorbable and non-absorbable suture material (Vicryl 4-0, Ethicon, Norderstedt, Germany and Resolon 4-0, Resorba Medical GmbH, Nürnberg, Germany). Wound surfaces were disinfected as previously described and treated with band aid spray (BSN Medical, Hamburg, Germany). Postoperatively, the animals were given novamine sulfone sodium (Ratiopharm GmbH, Ulm, Germany) via drinking water (0.03 ml/kg body weight) for analgesia for 1 week. The health status was also monitored for 1 week postoperatively and recorded in a score. Immediately after surgery, animals were able to move freely without restriction. Rabbits of the first group were sacrificed after 4 weeks, and those of the second group after 12 weeks. For this purpose, the triple dose of ketamine/xylazine per kg body weight was injected subcutaneously followed by 1 ml/kg body weight pentobarbital (release 500 mg/ml, WDT, Garbsen, Germany), which was administered intravenously. After completion of the sacrifice, both distal femora were harvested without damaging the region of surgery. Femora were collected in 50-ml tubes (Sarstedt AG & Co. KG, Nuembrecht, Germany), and condyles were separated from the shaft using a diamond-coated bone saw (EXAKT Advanced Technologies GmbH, Norderstedt, Germany). Those condyles, which were used for histological analysis, were layered with 4% phosphate-buffered formaldehyde (Grimm MED Logistik GmbH, Torgelow, Germany), and samples used for mechanical analysis were stored on ice.

### μCT analysis and biomechanical characterization

For μCT analysis, the proximal part above the condyles was removed using a diamond-coated bone saw. Subsequently, images of these samples were taken using the Skyscan 1172 μCT scanner (Bruker, Billerica, MA, USA) with the corresponding software. An aluminum filter (0.5 mm) was used to create the images, and the resolution was set to 17.2 μm in each direction. Afterward, images were reconstructed using NRecon 2.0 reconstruction software (Micro Photonics Inc., Allentown, PA, USA) and then visualized with Amira v6.4.0 (Thermo Fisher Scientific, Waltham, MA, USA). Following the μCT analysis, specimens of group 2 (12 weeks) underwent osteodensiometric analysis using the bone densitometer MEDIX DR (Diagnostic Medical Systems, Gallargues Le Montueux, France), whereas specimens of group 1 (4 weeks) were directly used for biomechanical evaluations. Prior to this, bone cylinders were removed from the defect site using a trepan drill (Ø 6 mm) under continuous rinsing with 0.9% sodium chloride as described before. The mechanical properties were analyzed using a uniaxial testing machine (Z050, ZwickRoell, Ulm, Germany) and a 2.5-kN load cell (ZwickRoell). The test was performed at room temperature. A preload of 0.1 N was applied, and afterward, the specimens were loaded with 0.05 mm/s until failure [12]. Autologous and allogeneic bone samples were compared regarding the offset yield strength as indicator for the initial mechanical failure of the graft.

### Gene expression analysis

Previously biomechanically tested samples were subsequently used for gene expression analyses. For this purpose, samples were digested overnight with 1 ml of collagenase A (Roche Diagnostics GmbH, Mannheim, Germany) at 37 °C to isolate cells. Afterward, nondigestible components were removed and samples were centrifuged at 1,000*g* for 5 min. The resulting pellet was resuspended in 600 μl of lysis buffer, which was a component of the innuPrep RNA Mini Kit (Analytik Jena AG, Jena, Germany). Samples were incubated for 15 min at room temperature. Following this, RNA was isolated according to the manufacturer’s instructions and concentration was measured using a Tecan Reader Infinite 200 Pro microplate reader (Tecan Trading AG, Maennedorf, Switzerland) with the provided elution water as blank.

The isolated RNA was transcribed to cDNA using the High-Capacity cDNA Reverse Transcription Kit (Applied Biosystems, Foster City, CA, USA). RNA (100 ng) was used, and reverse transcript polymerase chain reaction (RT-PCR) was performed (thermocycler from Analytik Jena) applying the following protocol: 10 min at 25 °C, 120 min at 37 °C, and 15 s at 85 °C. Afterward, 20 μl of ribonuclease-free water was added to each sample, which was then stored at −20 °C for further use.

For quantitative PCR (qPCR), a master mix was prepared for each gene to be analyzed. An overview of the genes of interest with their primer pairs is shown in Table 2.

For the master mix, 0.5 μl of forward primer, 0.5 μl of reverse primer, 3 μl of distilled water (all: Sigma-Aldrich, St. Louis, MO, USA), and 5 μl of innuMIX qPCR DSGreen (Analytik Jena AG, Jena, Germany) were blended. cDNA (1 μl per sample) was then pipetted in duplicates into the well of a 96-well PCR plate, and 9 μl of the master mix was added. The plate was covered with foil and then placed in the device of the qPCR tower (Qtower 2.0, Analytik Jena AG, Jena, Germany) to run the following protocol: 2 min at 95 °C, 40 cycles of 5 s at 95 °C and 25 s at 60 °C. Data points with a CT value above 30 were not interpreted. To analyze the results, the ΔΔCt method was used. Therefore, the relative expression of mRNA was compared to the housekeeping gene glyceraldehyde-3-phosphate dehydrogenase (*GAPDH*), which was calculated by the equation ΔCt = Ct_target_ − Ct_housekeeping gene_. Data were related to cylindric samples of each rabbit collected on the day of surgery (% native bone). Those were stored at −80 °C until further analysis and should reflect the normal bone metabolism of the animals.

### Histological analysis

The preparation of bone samples for histological analysis was performed analogous to Dau et al. [19]. Bone samples were cut into halves and fixed in 4% phosphate-buffered formalin for 7 days. In brief, specimens were dehydrated by incubation in ascending alcohol (70%-80%-90%-96% for 24 h each and 100% for 48 h). The samples were subsequently decalcified in 20% EDTA (pH 7.2 to 7.4) (USEDECALC, Medite GmbH, Burgdorf, Germany) over a period of 4 weeks. Decalcified specimens were embedded in paraffin. Afterward, slices were processed with a micro-grinding system (EXAKT, Norderstedt, Germany) to a final thickness of 4 μm. Prior to staining, samples were subjected to an enzymatic antigen demasking with a combination of trypsin and ethyl-diamine-tetraacetate for 15 min at 37 °C. This was followed by an overnight staining at 4 °C with primary antibodies osteocalcin monoclonal antibody (Invitrogen, Waltham, MA, USA) and BMP-2 polyclonal antibody (Thermo Fisher Scientific, Waltham, MA, USA) using a 1:200 dilution. After a washing step with PBS, antibodies were counterstained with the EnVision Detections System Peroxidase/DAB, Rabbit/Mouse (Agilent, Santa Clara, CA, USA). Images of slices were taken with Zeiss Axio Imager 2 using the Axiocam Mrc5 with a 20× objective (resolution: 0.68 μm/pixel) (all: Zeiss, Oberkochen, Germany). Analysis of samples was performed using QuPath by selecting the implant region [region of interest (ROI)] in a first step [20]. Afterward, 3,3′-diaminobenzidine (DAB)-positive pixels were detected using the “positive pixel count” command, which was then displayed as percentage of ROI. This was done for sections of all animals and depicted with GraphPad Prism 9 as bars (GraphPad Software, Boston, MA, USA).

### Data presentation and statistical analysis

Data of osteodensiometric and biomechanical analysis as well as gene expression results are presented in box plots with 25% and 75% quartiles by using the GraphPad Prism Software 9 (GraphPad Software, Boston, MA, USA). Statistical analysis was performed using one-way and two-way analysis of variance (ANOVA) with Bonferroni’s post hoc test after verification of normal distribution. *P* values of ≤0.05 were considered significant. Histological data were analyzed using QuPath v0.4.3 [20].

## Results

### Analysis of bone graft integrity

**Fig. 1.**
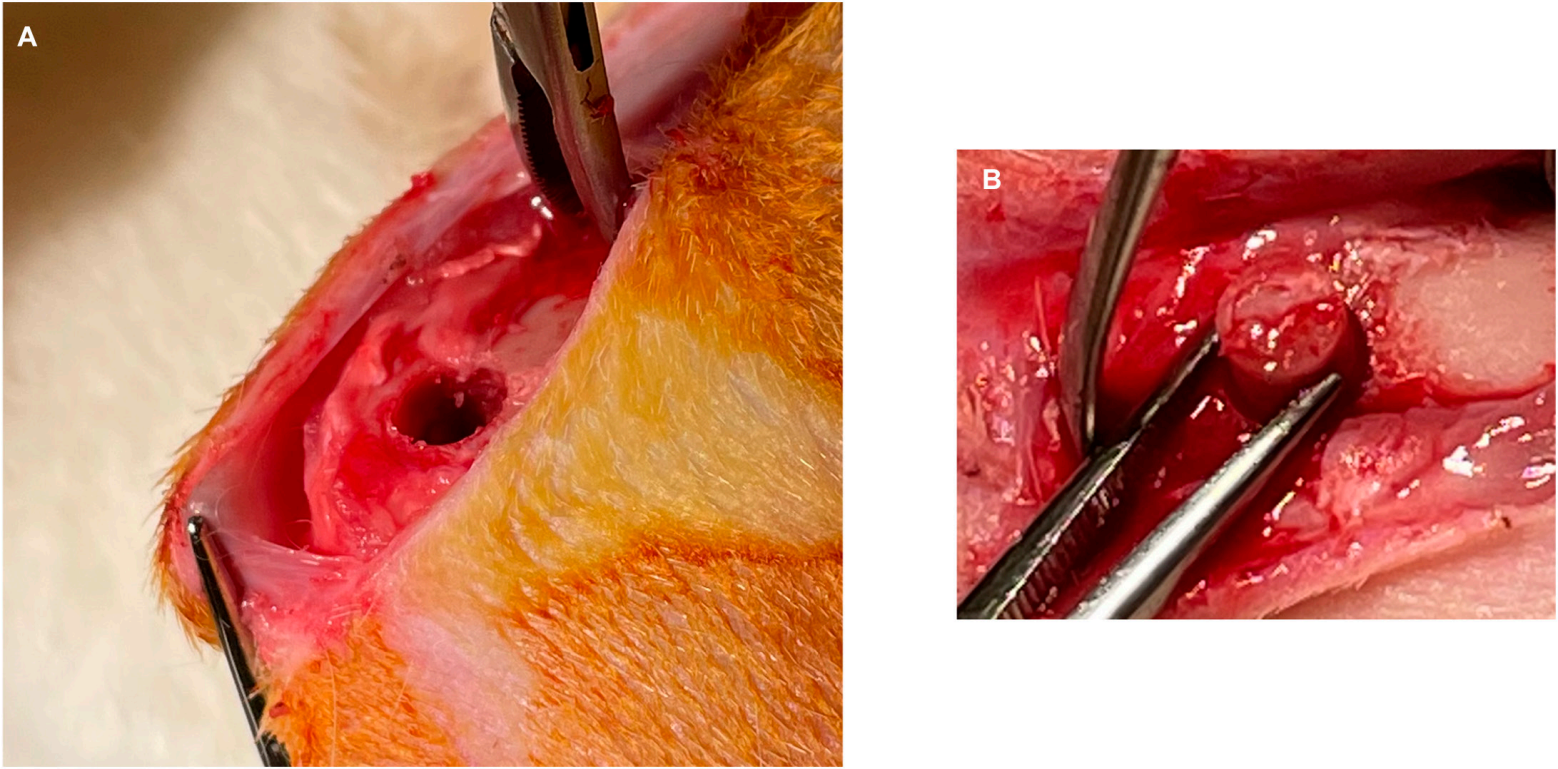
Surgical procedure. (A) Artificial bone defect in the lateral condylus with a diameter of 6 mm. (B) Insertion of the graft into the defect zone.

A total of 14 New Zealand White rabbits underwent the previously described surgery, in which a bilateral defect in the distal femoral condyles was set. In Fig. 1, images of the surgical procedure show the defect of the lateral condylus generated by the use of a trepan drill (Fig. 1A) and the insertion of the graft into the defect (Fig. 1B).

In order to assess the ingrowth behavior of the grafts into the surrounding bone, μCT analyses, histology, and bone mass density (BMD) analyses were performed.

**Fig. 2.**
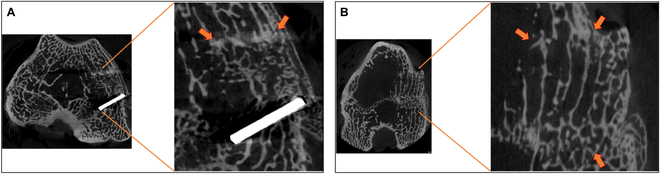
μCT images of rabbit condyles 4 weeks after implantation, with arrows indicating graft–bone interfaces. (A) Untreated autograft with tantalum sticks framing the graft. (B) HHP-treated allograft. After sacrifice of animals, femora were collected and condyles were separated from the femoral shaft. Samples were stored in 4% paraformaldehyde and underwent μCT imaging using Skyscan 1272, aluminum filter, and 17.2-μm resolution. Data were reconstructed using NRecon software and evaluated using Amira software. The edges of the graft were labeled with orange arrows.

Figure 2 shows representative images of rabbit condyles 4 weeks after surgery. For both, the autograft and the allograft, a connection of trabeculae of surrounding bone to trabeculae of the graft could be detected. By having a look at the autograft, a light emission of the upper tantalum stick was observed, which leads to artifacts in other levels of the tissue. Furthermore, it is noticeable that in case of the allograft, an optimal transition between the graft surface and the bone surface could not be achieved, which can be seen at the protruding edge in the upper right-hand corner of the right image. Nevertheless, the conjunction of the inner trabeculae was obtained.

**Fig. 3.**
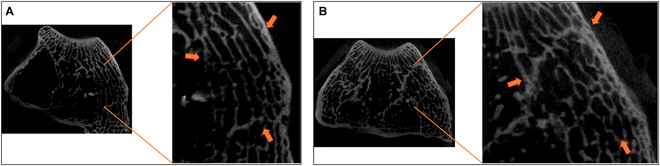
μCT images of rabbit condyles 12 weeks after implantation, with arrows indicating bone–graft interface. (A) Untreated autograft. (B) HHP-treated allograft. After sacrifice of animals, femora were collected and condyles were separated from the femoral shaft. They were stored in 4% formaldehyde and underwent μCT imaging using Skyscan 1272, aluminum filter, and 17.2-μm resolution. Data were reconstructed using NRecon software and evaluated using Amira software.

In Fig. 3, μCT images of rabbit condyles, 12 weeks after implantation, are depicted. Again, a connection of graft trabeculae with the surrounding bone was observed, whereas a distinction between graft and original bone was not possible anymore. Similar to images 4 weeks postoperatively, artifacts of tantalum sticks could be detected.

**Fig. 4.**
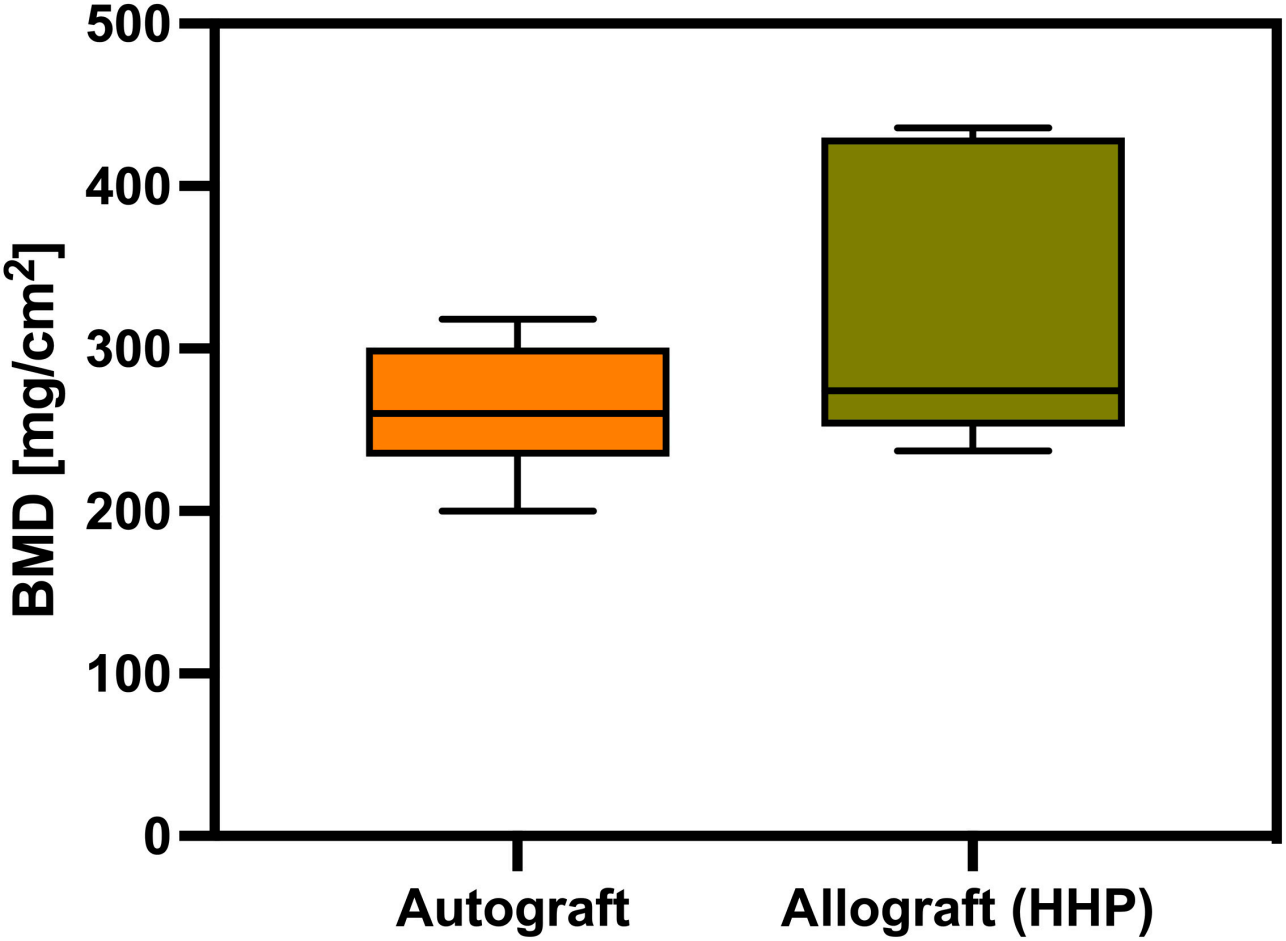
BMD analysis of explanted autograft and HHP-treated allografts 12 weeks after implantation. After sacrifice of animals, femora were collected and BMD of condyles was measured using a bone densitometer. Statistical analysis was performed using an unpaired *t* test (*n* = 5).

In addition to μCT images, the BMD of femoral condyles was measured for femora of 2 (12 weeks after surgery). The results are shown in Fig. 4.

The mean value for both autografts and HHP-treated allografts was very similar with approximately 270 mg/cm^2^. While standard deviation for autograft was comparatively low, a shift toward higher bone densities was detected for allografts. Despite this shift, no significant differences between bone autografts and allografts could be detected.

### Mechanical characterization of autografts and HHP-treated allografts after implantation

**Fig. 5.**
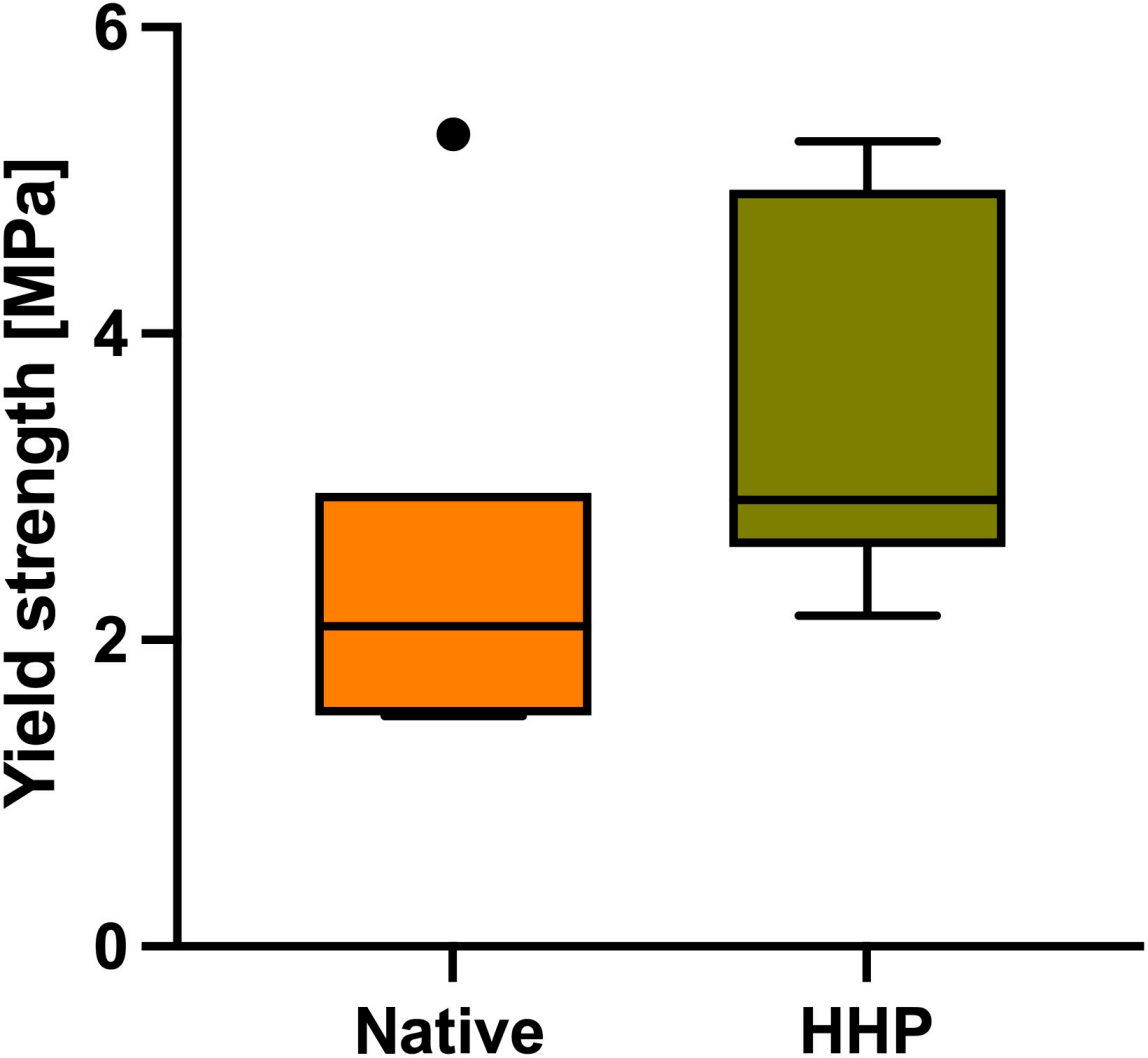
Yield strength of autografts and processed allografts after implantation of 12 weeks (*n* = 5). Following the sacrifice of the animals, cylinders of 6 mm in diameter and 10 mm in length were prepared from the side of surgery. A uniaxial compression test with a preload of 0.1 N and a test speed of 0.05 mm/s was performed. Yield strength of autografts and allografts was compared. Statistical analysis was performed using the Mann–Whitney *U* test.

Figure 5 shows the results of the uniaxial compression test of autografts and HHP-treated allografts after 4 and 12 weeks of implantation.

The yield strength of the explanted samples (Fig. 5) showed no significant differences between the native autograft and the HHP-treated allografts 12 weeks postoperatively. However, for HHP-treated allografts, a slightly higher mean value for the yield strength could be observed compared to the native allograft. Mechanical analyses were also carried out 4 weeks after implantation, but only 3 samples could be analyzed for each treatment, which made a statistical analysis not appropriate. For untreated samples, a mean yield strength of 3.9 MPa with a standard deviation of ±2.1 MPa could be detected, whereas HHP-treated allografts showed a mean yield strength of 4.1 MPa with a standard deviation of ±0.7 MPa. Comparing these data with those of 12 weeks after implantation, a slight decrease in the yield strength of autografts and allografts was observed over time.

### Molecular biological evaluation of bone remodeling processes after graft implantation

**Fig. 6.**
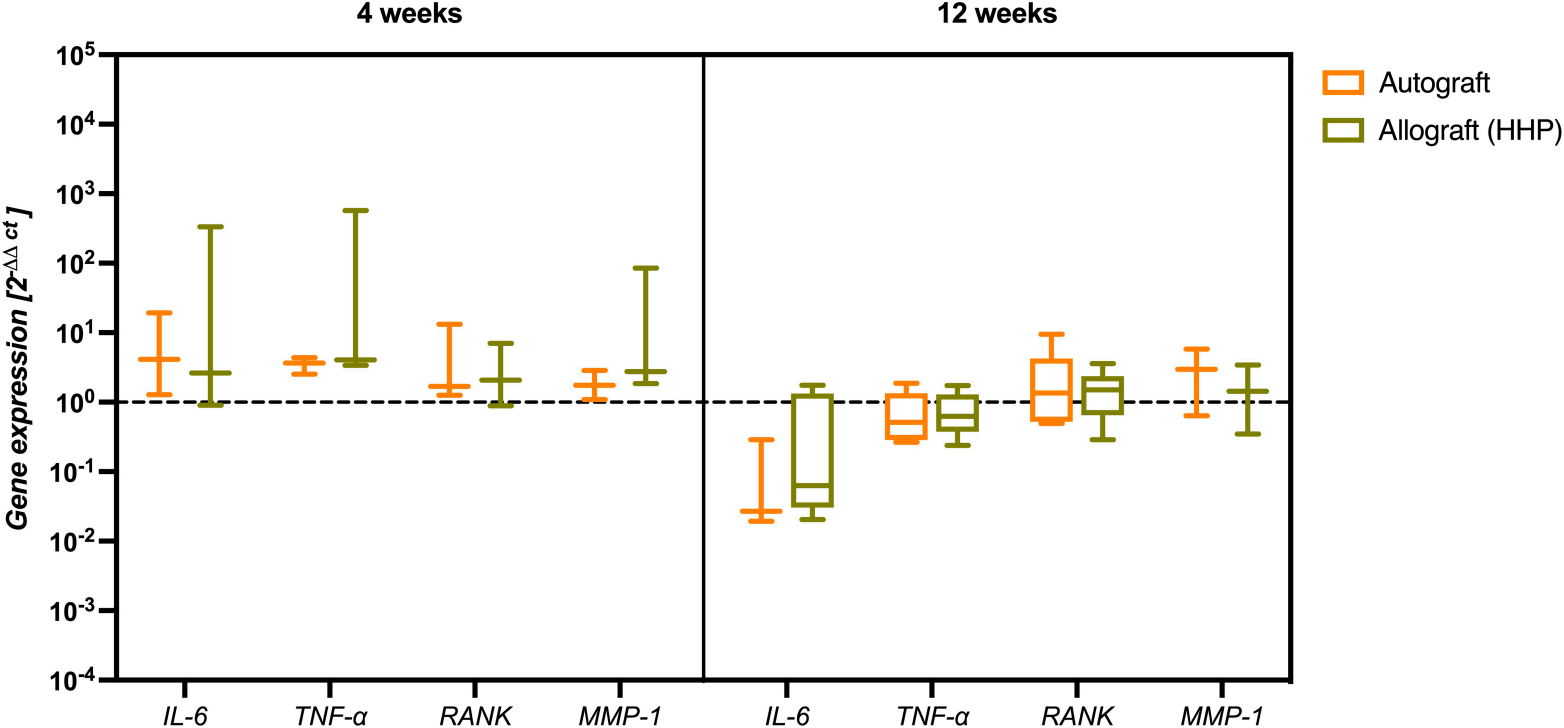
Gene expression of mediators associated with inflammation and bone resorption processes in autografts and HHP-treated allografts after 4 weeks (*n* = 3) and 12 weeks (*n* = 5) of implantation. Statistical analysis was performed using a two-way ANOVA with Bonferroni’s post hoc test. Dotted line depicts gene expression niveau of bone specimens explanted on the day of surgery, which were replaced by the allograft.

**Fig. 7.**
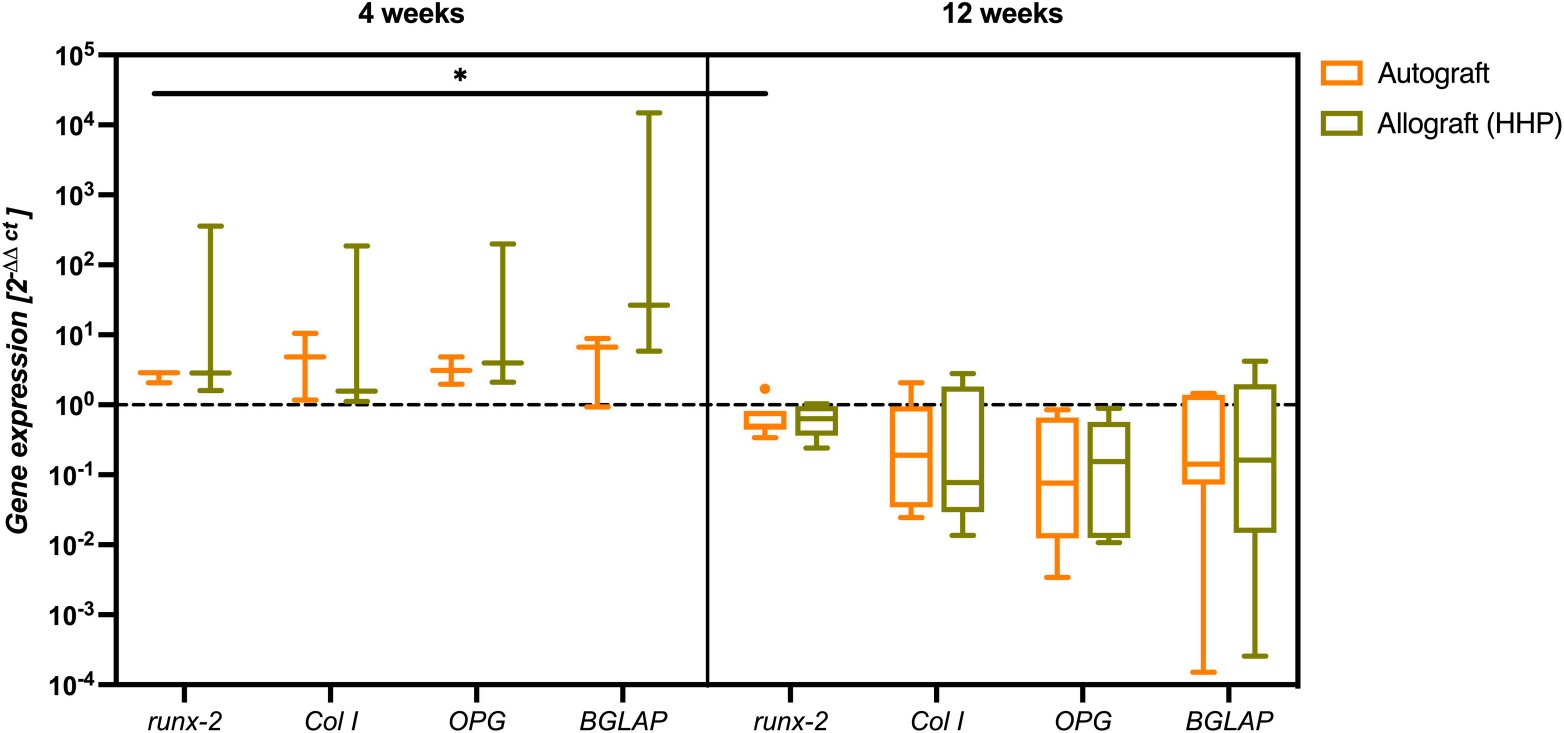
Gene expression of mediators associated with bone formation processes and osteogenic differentiation in autografts and HHP-treated allografts after 4 weeks (*n* = 3) and 12 weeks (*n* = 5) of implantation. Statistical analysis was performed using a two-way ANOVA with Bonferroni’s post hoc test (*P ≤ 0.05). Dotted line depicts gene expression level of bone specimens explanted on the day of surgery, which were replaced by the allograft.

In addition to biomechanical analysis and μCT imaging, autologous and allogeneic bone grafts were also analyzed regarding the expression of various mediators characteristic for bone metabolism. Results of gene expression analysis of mediators associated with bone resorption are shown in Fig. 6, and results of gene expression analysis of those associated with bone formation are shown in Fig. 7.

By having a look at the gene expression data of mediators, associated with bone resorption processes (Fig. 6), no significant differences between autografts and HHP-processed allografts could be observed. However, a slight increase in expression of *IL-6*, *TNF-α*, and *RANK* compared to the expression level of the explanted graft (dotted line) could be detected, but to a nonsignificant degree. After 12 weeks of implantation, a decrease in expression of *IL-6*, *TNF-α*, and *RANK* could be found, whereas *IL-6* and *TNF-α* were down-regulated below the control level and *RANK* expression was on the same niveau as the control. Also, mRNA transcripts of matrix metalloproteinase-1 (MMP-1) showed a down-regulation after 12 weeks compared to 4 weeks of implantation, but were still overexpressed in comparison with the original expression.

As depicted in Fig. 7, 4 weeks after implantation, an overexpression of mediators associated with bone formation compared to the original state of the bone could be observed for both groups. However, no significant differences between the expression of autografts and allografts could be observed. While the expression of Col I was lower in HHP-treated allografts compared to the autograft, allografts expressed slightly more BGLAP mRNA transcripts than autografts. Twelve weeks after implantation, autografts showed a significant decrease in runx-2 expression. Although this decrease in expression below the original expression level could be detected for all mediators, no statistical significance could be determined. In addition, no differences between the allograft and the autograft in the expression of runx-2, Col I, OPG, and BGLAP could be found.

### Histological evaluation of the bone grafts

**Fig. 8.**
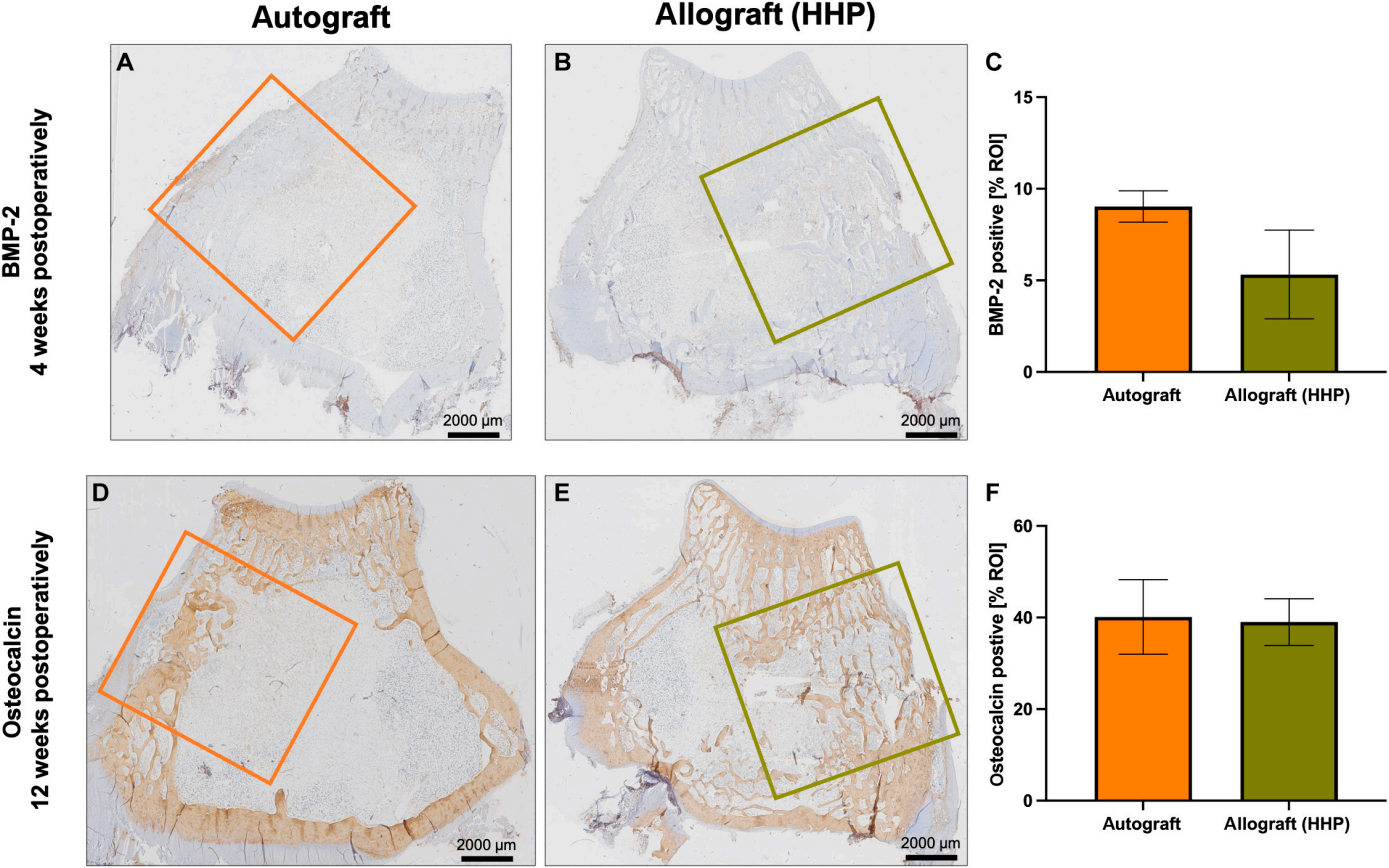
Representative images of BMP-2 (A and B) and osteocalcin (D and E) staining after 4 or 12 weeks postoperatively. In (A) and (D), autografts are depicted, whereas in (B) and (E) allografts are shown. Orange and green boxes represent the regions of interest (ROI), which represent the implant area and serve as the basis for calculating DAB-positive pixel count. Results of BMP-2 and osteocalcin positivity as % of ROI are shown in (C) and (F), respectively. Values were averaged from 3 images each and depicted as columns with standard deviation. Statistical analysis was performed using the Mann–Whitney *U* test (*n* = 3).

Histological analyses were performed to detect bone morphogenetic protein-2 (BMP-2), a mediator of osteoblast differentiation, as well as osteocalcin, a bone matrix protein. Results of histological staining and their evaluation are shown in Fig. 8.

Figure 8 shows representative images of histological staining of BMP-2 (4 weeks after implantation) and osteocalcin (12 weeks after implantation). It is striking that BMP-2 appears much weaker than osteocalcin, although the protocol was the same for both antibodies. Nevertheless, a DAB-positive pixel count in the ROI for both autograft and allograft could be detected, which was averaged from 3 images each (Fig. 8C). While for the autograft about 8% BMP-2-positive pixels of the ROI could be detected, the allograft shows slightly less BMP-2-positive pixels with approximately 6% (Fig. 8C). Yet, no significant differences between autograft and allograft could be detected. This was also shown for osteocalcin-positive areas: 12 weeks after implantation, autograft and allograft did have almost similar amounts of osteocalcin-positive pixels with approximately 39% (Fig. 8F). Hence, no significant differences between autograft and allograft could be observed.

## Discussion

With more than 2 million surgeries performed worldwide each year, bone tissue is the second most commonly used transplant right after blood [21]. For defect reconstruction, autologous bone is still considered as gold standard due to its osteoinductive and osteoconductive properties and low probability of rejection [22]. Allogenic bone grafts, which are more easily accessible than autologous bone, lack the aforementioned properties as a result of complex chemical and physical decellularization processes [7]. In particular, biomechanical properties are significantly reduced as a result of graft processing [23]. Previous in vitro studies have already shown that HHP does effectively devitalize cancellous bone without causing structural damage while preserving the biomechanical properties of the bone [17]. Based on the in vitro results, the aim of the present analysis was to investigate whether the HHP-treated bone allograft is suitable for the reconstruction of bone defects in vivo. As the application of HHP has so far only been approved for decontamination of food, the purpose of this study was to investigate the potential of the HHP for the preparation of bone grafts and thus the application for clinical use. Attention was paid on the presence of bone remodeling processes on the one hand and on biomechanical stability and integration of the allogeneic bone graft on the other hand.

Four weeks after surgery, μCT images revealed the beginning of integration of the graft into the surrounding tissue. At the edges of the transplant, a connection between adjacent trabeculae could be recognized, whereby no differences in the degree of ingrowth between autograft and allograft could be observed. Such early integration of bone transplants into the adjacent bone was already observed in other studies, e.g., the study of Dumas et al. [24] showed histological evidence of bone remodeling processes and bone formation after 4 weeks postoperatively. Furthermore, Park et al. [25], who also chose μCT for evaluation, observed first hints of bone integration 2 weeks postoperatively whereby original bone and implant could be clearly distinguished due to small gaps. By having a closer look at the 12-week time point, μCT analysis indicated the entire union between bone graft and surrounding bone to such an extent that a distinction between original bone and the graft was no longer possible. Similar results were obtained by Rastegar et al. [26], whose study focused on decellularized, xenogeneic extracellular matrix as bone replacement material. Twelve weeks postoperatively, only the empty defect showed low rates of bone remodeling resulting in a still existing defect, whereas all other groups confirmed good graft integration. Such a comparison of the ingrowth behavior of HHP-treated allografts versus the empty defect could not be carried out in the study at hand, as the replacement of the defect with autologous bone was selected as reference. It is well known from a variety of other studies that the pure injury heals worse than the defects in which bone replacement materials were inserted [27]. Taking into account the size of the defect that is considered critical in rabbits from a diameter of 3 mm and a depth of 3 to 5 mm, studies with an experimental setup similar to ours are always above the recommended values [28]. It can therefore be assumed that the used defect size of a diameter of 6 mm and 7 to 10 mm depth is also critical for spontaneous healing and that an empty defect would not be healed even after 12 weeks [29]. Therefore, the aim of this study was not to demonstrate the inferiority of the pure defect compared to a grafted one, but rather comparing the HHP-processed allograft with the autograft, which is known to offer the best clinical outcome [30].

Furthermore, 12 weeks postoperatively, Park et al. [25] showed successful integration of cortical grafts, which underwent different sterilization procedures. Similar to Park et al., the grafts in our present study were equipped with metal pins with the idea of localizing the implant by μCT at day of sacrifice. While this was still possible for 4 weeks after implantation, it turned out to be unsuitable for the 12-week group as the tantalum pin migrated through the bone over time. The study of Lee et al. [31], which also used tantalum pins in their in vivo study, did not demonstrate such a problem even after 16 weeks of implantation. However, these pins were significantly larger than those used in our present study (diameter of 2 mm, length of 10 mm). Furthermore, access to the bone took place through the trochlear groove and inserted pins reached the bone marrow, representing a different surgical situation [31]. It can be assumed that migration of the pins was due to bone remodeling processes, which were probably promoted by the bony grafts that the tantalum pins were surrounded by. Due to the small size, a free movement in all directions was possible, resulting in the change of location. In order to better track the grafts in future in vivo studies and possibly observe neovascularization at the same time, the use of dynamic contrast-enhanced magnetic resonance imaging introduced by Righesso et al. [32] could be a suitable method. However, the animals would have to be anesthetized sequentially over time, which creates additional stress to them. Furthermore, rabbits are more prone to anesthesia than other small animals, and so such interventions should be kept to a minimum [33].

The presence of the previously mentioned bone remodeling processes was evaluated primarily by gene expression analysis. At this point, it should be mentioned that individual samples scatter very widely, so large standard deviations were detected. This reflects the variability of biological samples and is not unusual for studies including individuals [34]. Since analyses does only consider the expression of mRNA, no statements can be made about the presence of proteins and the integrity or functionality of the graft [35]. Therefore, it is only utilized as a supporting parameter for other data collected in this study. In these experiments, the expression patterns of mediators associated with bone resorption (*IL-6, TNF-α, RANK, MMP-1*) as well as mediators associated with bone formation (*runx-2, Col I, OPG, BGLAP*) were focused [36]. It is known that bone resorption and formation must occur in precise balance for remodeling to be successful [37]. Although it is desirable for bone substitute materials to be degradable, the resorption rate must not be too high as this would lead to a failure of the graft [22]. In the study at hand, it could be shown that no significant differences between the expression of the mentioned mediators were observed, comparing autologous bone with HHP-treated bone allografts. Four weeks after surgery, an up-regulation of expression of mRNA transcripts was noticed for both mediators of bone resorption and bone formation. The comparison was drawn to the bone, which was removed at the day of surgery, as this should represent the status quo of a healthy bone metabolism. It can therefore be assumed that bone remodeling processes were induced by the creation of the defect as well as the insertion of the graft, resulting in the increased expression of the mentioned mediators. However, it should also be noted that resorption and formation are balanced as the mRNA transcripts are expressed in the same range. This balance could also be determined for all mediators even after 12 weeks of implantation, whereby for all a down-regulation in expression was observed, in some cases below the initial level. This leads to the assumption that bone remodeling processes have been completed, for which also a hint could be found having a look at the μCT images showing the comprehensive integration of the graft. A former study by Virolainen et al. [38] also focused on mRNA expression levels and observed peaks in mRNA transcript expression on day 5 or 7 (procollagen and collagen) or on day 14 (osteonectin) after transplanting autografts and allografts. Twenty-eight days after surgery, a drop in mRNA expression was shown and was again significantly reduced on day 56 after implantation [38]. Since first samples were taken after 28 days in our present study, it may not have been possible to detect particularly high mRNA expression levels, as acute remodeling processes might be already completed [38]. This also indicates that mediators of bone resorption, which are temporally expressed before mediators of bone formation, should be observed earlier than day 14 of transplantation [39]. However, it is difficult to compare these results with other studies. Histomorphometric analyses are often used to assess bone remodeling processes, e.g., by determining the number of osteoblast and osteoclast-like cells, which could provide information about active bone metabolism. However, for these analyses, the graft–host interface must be clearly recognizable. This is why histomorphometry is often the method of choice for studies in which this interface is visible due to the implantation of materials that differ from native bone, coatings of the inserted bone, or the formation of callus [40]. Due to the high similarity of grafts chosen in this study and the region of transplantation, an exact distinction between graft and host is not possible, which is represented in the histological images. Nevertheless, by selecting regions of interest (ROI) of the same size, an analysis could be carried out with regard to the BMP-2 distribution and the osteocalcin deposition in the graft region. Compared to osteocalcin, BMP-2 staining was relatively weak. Since BMP-2 promotes bone formation and contributes to osteogenic cell differentiation, it should be able to be detected in the early phases of bone formation [41]. With this knowledge, it is striking that the staining is such weak. BMP-2 expression goes in course of differentiation hand in hand with collagen expression [42]. According to the study of Virolainen et al. [38], which showed already reduced expression of collagen at 28 days of implantation, the reduced expression of BMP-2 in histological staining is no longer surprising. It can be assumed that the expression of BMP-2 took place at earlier time points, which were not depicted in this study. Although the time points chosen here for final analyses are not unusual for animal studies including rabbits, the aspects observed in this work indicate a very rapid bone metabolism in these animals [26]. For further studies, it would therefore be conceivable to focus on earlier time points for analyses, e.g., 2 weeks after surgery.

A strong osteocalcin-positive staining 12 weeks postoperatively for both implants was observed, and for BMP-2, no significant difference between autograft and allograft could be detected. Osteocalcin comprises 10 to 20% of the extracellular bone matrix and is known as a late marker of bone formation [43]. The results presented here show a broad distribution of osteocalcin across the entire section after 12 weeks of implantation, which has already been observed in other studies with similarly long postoperative periods [44]. Besides the critical role for bone formation processes, osteocalcin does also contribute to the mechanical strength of the bone [43]. Biomechanical properties, detected after 12 weeks of implantation by performing a uniaxial compression test, choosing yield strength as parameter of interest, showed no significant differences between autograft and HHP-treated allograft. With a mean value of 2.7 ± 1.4 MPa, these values are comparable to those of healthy, untreated bones [45]. Furthermore, the grafts used in this study on hand also performed significantly better than demineralized bone matrices, which only showed an average yield strength of 1.3 ± 0.5 MPa after 60 days of implantation [45]. It can be concluded that HHP does not negatively influence the biomechanical properties of cancellous bone neither in vitro, as already been described, nor after implantation in vivo [12]. As mentioned before, no significant differences between the yield strength of autografts and HHP-treated allografts were observed. Nevertheless, bone allografts showed slightly higher values than autografts. This was also observed for measurements of bone mineral density (BMD) by Haba et al. [46], who analyzed the correlation of biomechanical properties and BMD. However, by comparing the BMD found in our study with the BMD data of untreated rabbits measured by Castañeda et al. [47], a difference of approximately 155 mg/cm^2^ is registered. According to Castañeda et al. [47], the values determined in this study on hand are more in line with that of osteoporotic animals. However, the work by Castañeda et al. [47] analyzed mainly subchondral areas, while this study focused on the entire femoral condyles, which could explain the differences. A reliable statement about bone structure would not be possible with BMD alone. Therefore, peripheral quantitative computed tomography (pQCT) before surgery is recommended, with which individual differences can be taken into account [48]. Besides the individual differences, it is also important to point out the differences between rabbits and humans, which makes translation to clinical applications challenging. Due to anatomical and physiological reasons, rabbits have less cancellous bone and more fragile cortical bone than humans [48]. Furthermore, differences in bone microstructure as well as bone composition were observed. However, rabbit bone metabolism shows a similarity to human bone remodeling due to the presence of secondary osteochondral remodeling [49]. The choice of adult animals also ensures that excessive regeneration, as it occurs in young animals, does not influence the remodeling process [48]. With regard to the fact that bone healing in rabbits is 3 times faster than in humans, the selected time points “4 weeks” and “12 weeks” after surgery are adequate to represent an early and a late stage of regeneration for both species [49]. Nevertheless, only applications of HHP-treated bone grafts within a clinical study with humans can demonstrate effective performance of the graft.

The behavior of HHP-treated cancellous bone allografts regarding mechanical properties and integration into the surrounding bone was evaluated in vivo and compared to bone autografts. We could show that processed allografts and untreated autografts did not significantly differ from each other regarding bone metabolism, biomechanical integrity, or deposition of extracellular matrix proteins. Hence, it can be concluded that the HHP treatment protocol used for devitalization does not negatively influence the integration of the processed allogeneic bone grafts.

## Data Availability

The data that support the findings of this study are available from the corresponding author (J.W.-H.) upon reasonable request.
