# Supplementary material for: Evaluation of Integrity of Allogeneic Bone Processed with High Hydrostatic Pressure: A Pilot Animal Study
Source: Biomater Res. 2024 Aug 15;28:0067. doi: 10.34133/bmr.0067 (PMC11325089; doi:10.34133/bmr.0067)
Supplement: Supplementary 1 — Tables S1 and S2 [file bmr.0067.f1.zip › Table 1.docx]

Table 1: Overview of number of animals, life time after surgery and analysis of samples after sacrifice. BMD – bone mass density

| Number of animals | Postoperative life time | Analysis |
| --- | --- | --- |
| 3 | 4 weeks | µCT / histology |
| 3 | 4 weeks | gene expression analysis |
| 3 | 12 weeks | µCT / histology |
| 5 | 12 weeks | Gene expression analysis / biomechanical analysis / measurement of BMD |
