# Supplementary material for: Evaluation of Integrity of Allogeneic Bone Processed with High Hydrostatic Pressure: A Pilot Animal Study
Source: Biomater Res. 2024 Aug 15;28:0067. doi: 10.34133/bmr.0067 (PMC11325089; doi:10.34133/bmr.0067)
Supplement: Supplementary 1 — Tables S1 and S2 [file bmr.0067.f1.zip › Table 2.docx]

Table 2: Gene list with the corresponding forward and reverse primers used in the present study.

| Gene | Forward primer (5’ – 3’) | Reverse primer (5’ – 3’) |
| --- | --- | --- |
| *IL-6* | GCGGTGAATAATGAGACCTGC | TCGTCACTCCTGAACTTGGC |
| *MMP-1* | TGATGTGGCTCAGTTCGTCC | TTGTCACGATGATCTCCCCTG |
| *TNF-α* | GTGACGAGCCTCTAAGCCCACGTA | GACCGCTGAAGAGAACCTGGGAG |
| *RANK* | TTCAGGTTTGCTGTTCCTACAA | CGCCGTTTTATCCTCTCTACAC |
| *Runx-2* | TGATGACACTGCCACCTCTGA | GCACCTGCCTGGCTCTTCT |
| *Col I* | TCGATGGCTGCACGAAAAAG | AGGAAGGGCAAACGAGATGG |
| *OPG* | ACAATGAACAAGTGGCTGTGCTG | CGGTTTCTGGGTCATAATGCAAG |
| *BGLAP* | GCACAGAGCGACAGCATGA | CTCTTGGACACGAAGGCTGA |
| *GAP-DH* | CCACTTTGTGAAGCTCATTTCCT | TCGTCCTCCTCTGGTGCTCT |
